# 0020. Microcirculatory perfusion and vascular reactivity are altered in post cardiac arrest patients, irrespective of target temperature management to 33° vs 36° (substudy TTM)

**DOI:** 10.1186/2197-425X-2-S1-O1

**Published:** 2014-09-26

**Authors:** M Koopmans, MA Kuiper, R Endeman, G Veenstra, NAR Vellinga, R Vos de, EC Boerma

**Affiliations:** Medical Center Leeuwarden, Intensive Care, Leeuwarden, Netherlands; Onze Lieve Vrouwe Gasthuis, Intensive Care, Amsterdam, Netherlands; Academic Medical Center, Epidemiologic, Biostatistics and Bioinformatics, Amsterdam, Netherlands

## Introduction

After cardiopulmonary resuscitation (CPR), following an out of hospital cardiac arrest (OHCA) hemodynamic failure is common, due to a combination of heart failure and ischemia reperfusion injury. Comatose post-cardiac arrest patients are treated on the intensive care unit (ICU) with mild therapeutic hypothermia (33°), nowadays referred to as target temperature management (TTM) for an assumed neuroprotective effect.

## Objectives

In previous reports both microcirculatory alterations and impaired vascular reactivity have been described in post cardiac arrest patients treated with mild therapeutic hypothermia. As of now it is unknown whether these alterations are related to the temperature management itself. Aim of the present study was to investigate the potential difference in microcirculatory alterations and vascular reactivity in patients after out of hospital cardiac arrest treated with target temperature management of 33°C (TTM33) in comparising to patients treated with 36°C (TTM36).

## Methods

Our study was designed as a a priory substudy of the open label randomized controlled TTM trial in 2 Dutch mixed ICU's. Microvascular flow index (MFI) was assessed by Side Stream Darkfield imaging and vascular reactivity by near infrared spectroscopy. Variables, including systemic haemodynamics were recorded at start study (T1), after 12 hours (T2) and after 24 hours (T3).

## Results

22 patients were included, 13 in TTM33 and 9 in TTM36. At T1 MFI between groups did not differ significantly (1.08[0.4-1.9] versus 1.67[0.7-2.4] respectively, p=0.59) . The difference between groups remained insignificant over time. At T1 tissue oxygenation (StO2) was significantly lower in TTM36 in comparison to TTM33 (58.9±13.5 versus 44.6±15.8, p=0.03). Over time this difference between groups disappeared. However, vascular reactivity, expressed as the descending and ascending slope of StO2 after a standardized ischemic occlusion test was similar between groups

## Conclusions

In this relatively small sample size study microcirculatory blood flow and vascular reactivity did not change between TTM33 and TTM36

Clinicaltrials.gov nr. NCT01850485
